# Retraction Note: The significance of the change pattern of serum CA125 level for judging prognosis and diagnosing recurrences of epithelial ovarian cancer

**DOI:** 10.1186/s13048-022-01008-x

**Published:** 2022-07-01

**Authors:** Zhi-jun Yang, Bing-bing Zhao, Li Li

**Affiliations:** Department of Gynecologic Oncology, Affiliated Tumor Hospital of Guangxi, Medical University, 71# Hedi Road, Nanning, Guangxi 530021 People’s Republic of China

**Retraction Note: J Ovarian Res 9, 57 (2016)**

10.1186/s13048-016-0266-3

The Editor-in-Chief has retracted this article. After publication, the authors informed the journal that the associated data and documents were lost to technical issues. Therefore, the authors are no longer able to provide the raw data or evidence of ethics approval and patient consent.

None of the authors have responded to any correspondence from the editor or publisher about this retraction notice.

